# Control of a Drone in Virtual Reality Using MEMS Sensor Technology and Machine Learning

**DOI:** 10.3390/mi13040521

**Published:** 2022-03-26

**Authors:** Florin Covaciu, Anca-Elena Iordan

**Affiliations:** 1 Department of Design Engineering and Robotics, Technical University of Cluj-Napoca, 400641 Cluj-Napoca, Romania; florin.covaciu@muri.utcluj.ro; 2 Department of Computer Science, Technical University of Cluj-Napoca, 400027 Cluj-Napoca, Romania

**Keywords:** MEMS sensors, machine learning, virtual reality, accelerometer, magnetometer, gyroscope, sensors fusion

## Abstract

In recent years, drones have been widely used in various applications, from entertainment, agriculture, their use in photo and video services, military applications and so on. The risk of accidents while using a drone is quite high. To meet this risk, the most important solution is to use a device that helps and simplifies the control of a drone; in addition, the training of drone pilots is very important. To train the drone pilots, both physical and virtual environments can be used, but the probability of an accident is higher for beginners, so the safest method is to train in a virtual environment. The aim of this study is to develop a new device for controlling a drone in a virtual environment. This device is attached to the upper limb of the person involved in the control of that drone. For precise control, the newly created device uses MEMS sensor technology and artificial intelligence-specific methods.

## 1. Introduction

To identify human behavior, it is possible to facilitate the development of the human body’s sensor network with the help of several flexible devices in different anatomical locations. Additionally, with the expansion of perceptions, the rapid development of modern society has witnessed an increase in daily interaction for wearable electronic applications. Portable electronic devices have features that include measuring pressure, temperature, electrophysiology, blood oximetry, position, acceleration, magnetic field and so on. Due to the development of the human body sensor network for human behavior between humans and machines, the help of several flexible devices in different anatomical locations can lead to huge requirements of intelligent human–machine interfaces. A future sensor that uses machine learning promotes the trend of developing next-generation intelligent detection around big data for supersensitive detection [1]. Virtual reality (VR) or augmented reality (AR) technology has become a hot topic of research with the rapid development of the information industry and human–machine interface technology in recent years when combined with portable devices to form an entire interactive system in 3D space. In many application scenarios, this type of interactive system provides users with a more exciting experience with future sensors and microelectromechanical systems (MEMS)/nanoelectromechanical systems (NEMS) [2].

The development of services and applications involving drones has been greatly promoted with the growth of the unmanned aerial vehicle industry. Moreover, many industries, such as the entertainment industry, use drones to perform tasks that are difficult to perform by conventional methods.

By using virtual reality technology, a drone flight environment can be created that mimics a real space and, in this space, we can do drone control trainings. In such an environment, even beginners can safely practice drone flying. For example, accidents are no longer possible in this virtual reality environment.

This paper proposes a remote training system for drone flying in a virtual reality environment. The main contributions of this study are the following:

- A remote training simulator for drone flight is proposed using virtual reality, developing a simulator in the Unity development environment. The drone is controlled remotely in the virtual reality simulator via the Wi-Fi protocol using a microcontroller development board that includes a Wi-Fi transmission module. MEMS sensor technology is used for precise drone control, and a bracelet has been built for this purpose, which includes a microcontroller and a sensor module with which the user can control the drone.
- For the connection of the bracelet to the computer, a user interface was developed in the Visual Studio development environment, using the C Sharp (C#) programming language. Additionally, through this user interface, the connection with the virtual reality simulator is made.
- Specific methods of artificial intelligence, namely machine learning, are used to help control the drone. With the methods implemented by the Keras library [3] from TensorFlow, an MLP neural network with four inputs and three outputs was implemented to guide the movement of the drone.

## 2. Background

In the current literature can be found various training simulators for drone flight using virtual reality. These simulators mimic a real space, being used by people who want to train in these environments without crashing the drone during training. The simulators are used for various applications, using MEMS sensor technology in drone control. Artificial intelligence-specific methods are often used to help control a drone.

A recent example is the development of a virtual reality simulator that uses a wearable interface through which a human can team with multiple drones. Here, the authors introduced a virtual drone search game as a first step in creating a mixed reality simulation for people to practice search and rescue techniques and drone grouping. In this study, participants played the game in the virtual simulator using control through two input modalities: gestures and tap. The results showed that the participants who used gesture control performed best [1]. Virtual reality was also used to create a simulator, used to visually stimulate patients in lower limb rehabilitation applications [4,5]. Li et al. presents the design and development of a virtual reality-based system for training inspectors who are assisted by a drone in inspecting a bridge. This system consists of four modules, namely: a simulated bridge inspection developed in the Unity virtual reality program, an interface that allows a learner to operate the drone in simulation using a remote control, monitoring and analysis that analyzes the data to provide time real feedback for learners to help them learn and a post-study assessment to accelerate student learning [6]. 

In another study, researchers developed a system for collecting data on students’ educational development performance while using the virtual reality drone training simulator called Gamified and to objectively analyze student development. In this study, it was observed that the meditation and attention scores of the participants increased with the number of repetitions of the educational game, and thus it could be concluded that the number of repetitions decreases the level of stress and anxiety increases attention and, thus, improves game performance [7]. Nguyen et al. propose a prototype of a virtual reality application called DroneVR to eliminate problems in controlling a drone in the real environment, such as: drone crash due to uncontrolled environment and loss of signal that causes the drone to hit buildings in return mode [8]. Rognon et al. describe an exoskeleton called FlyJacket, designed for users who want to control a drone in a virtual reality application, with gestures for the upper body, in an intuitive way. When using this exoskeleton, the participants felt submerged, having a flight sensation, which proved that FlyJacket can be used for teleoperating a real drone [9]. The integration of Virtual Reality tools and Artificial Intelligence algorithms in medical applications was also demonstrated in [10], emphasizing the importance of these tools in the development of innovative systems able to work safely in human populated environments. In order to improve the accuracy of a gyroscope with a micromechanical system, noise modeling was performed using and evaluating an advanced deep recurrent neural network [11]. Rybarczyk presents preliminary research into the use of a MEMS accelerometer as a measuring element in the electrohydraulic control system [12]. 

Zhu at al. present a review paper which summarizes the development trends and perspectives of future sensors and MEMS/NEMS [1]. In [13], authors proposed a classification of a robot’s condition using a MEMS gyroscope with a three-axis and machine learning methods. Following the analysis of the learning results of the classifier, the possibility of using the k-nearest neighbor algorithm to classify the robot’s condition with an accuracy of 88% was shown. Xing et al. proposed the use of the wavelet filter to reduce the noise in the original MEMS gyroscope data, after which to reconstruct the random drift data with the phase space reconstruction and establish the model for the reconstructed data by least squares support vector machine, of which the parameters were optimized using chaotic particle swarm optimization. After comparing the effect of modeling the random drift of the MEMS gyroscope with back propagation artificial neural network and the proposed method, the results showed that the proposed method had a better prediction accuracy [14]. 

In the context of the ageing global population, Tsinganos at al. propose a study comparing three fusion schemes that have been applied in recognizing human activity and detecting falls. These algorithms are also compared to a recent study by the authors on the detection of falls in which only one type of sensor is used. The results showed that a machine learning strategy should be preferred because fusion algorithms differ in their performance [15]. The concept of autonomous advanced systems with high interaction capability with external features is also used in fields such as robotic assisted rehabilitation [16] where the combined use of experimental data with Fuzzy logic algorithms [17] enables the development of innovative safe robotic devices successfully tested in clinical trials [18,19]. 

With the rapid growth of manufacturing techniques for micro and nanodevices, an endless range of possible applications has emerged, with these new products significantly improving human life. However, the evolution in the process of design, simulation and optimization of these products has not seen such rapid growth, becoming clear that the performance of micro and nanodevices will be able to benefit from significant improvements in this area. In this regard, Esteves et al. presents a new methodology for electromechanical co-optimization of inertial sensors of MEMS systems by developing a program that includes geometry design, finite element method analysis (FEM), amortization calculation, electronic domain simulation and a process for optimizing the genetic algorithm (GA). An open-source program with the Python programming language was used to develop this program, using dedicated libraries for this purpose [20]. 

## 3. Introduction to MEMS Sensors That Are Used in the Application

An absolute orientation sensor containing the IMO BNO055 [21] chip is used for this application. This sensor is developed by Bosch company, being a nine-axis sensor that contains an accelerometer, magnetometer and gyroscope. Using this type of sensor, one of the challenges can be to transform the data from the accelerometer, magnetometer and gyroscope sensor into the orientation of real 3D space. Orientation is a difficult problem to solve, and sensor fusion algorithms that combine accelerometer, magnetometer and gyroscope data into a stable three-axis orientation output can be extremely difficult to correct and implement in low-cost real-time systems [22]. Bosch is the first company to achieve this, taking a MEMS accelerometer, magnetometer and gyroscope and putting them on a single matrix with a high-speed ARM Cortex-M0 processor to process all sensor data, abstract sensor fusion and requirements in real time and obtain data that we can use in quaternions, Euler angles or vectors. The BNO055 is a system in package, integrating a triaxial 14-bit accelerometer, a triaxial 16-bit gyroscope with a range of ± 2000 degrees per second and a triaxial geomagnetic sensor. Operating condition: supply voltage 2.4–3.6 V, operating temperature −40 + 85 °C. The characteristics of the three sensors are as follows:

*Accelerometer*: Programmable functionality—Acceleration range: ±2 g/±4 g ±8 g ±16 g; Low-pass filter bandwidth 1 kHz—<8 Hz.

*Gyroscope*: Programmable functionality—Ranges switchable from ±125°/s to ±2000°/s; Low-pass filter bandwidths 523–12 Hz.

*Magnetometer*: Flexible functionality—Magnetic field range typical ±1300 µT (x-, *y*-axis), ±2500 µT (*z*-axis); Magnetic field resolution of ~0.3 µT. 

**Figure 1:**
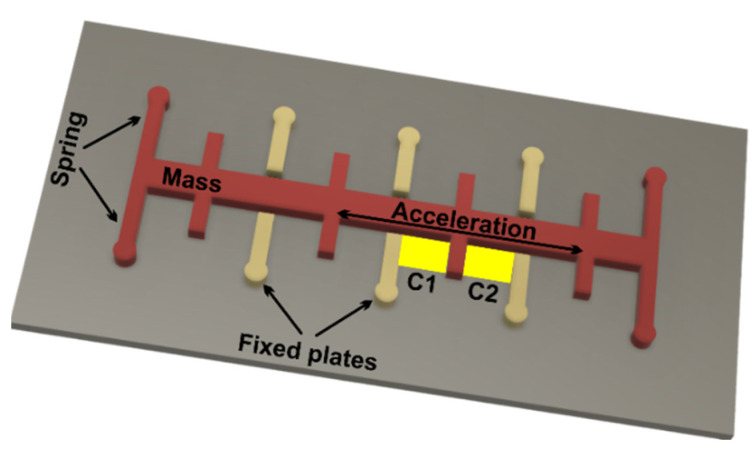
Microstructure of an accelerometer.

**Figure 2:**
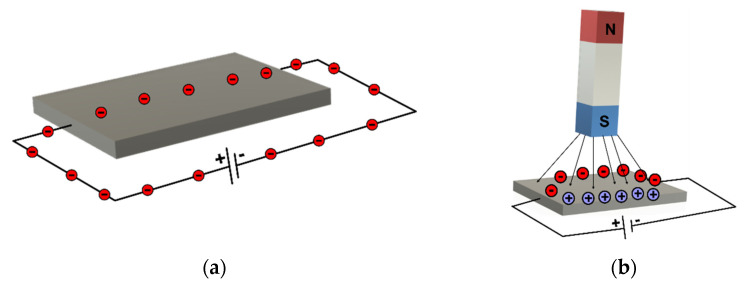
(**a**) MEMS magnetometer—(**b**) Hall effect.

**Figure 3:**
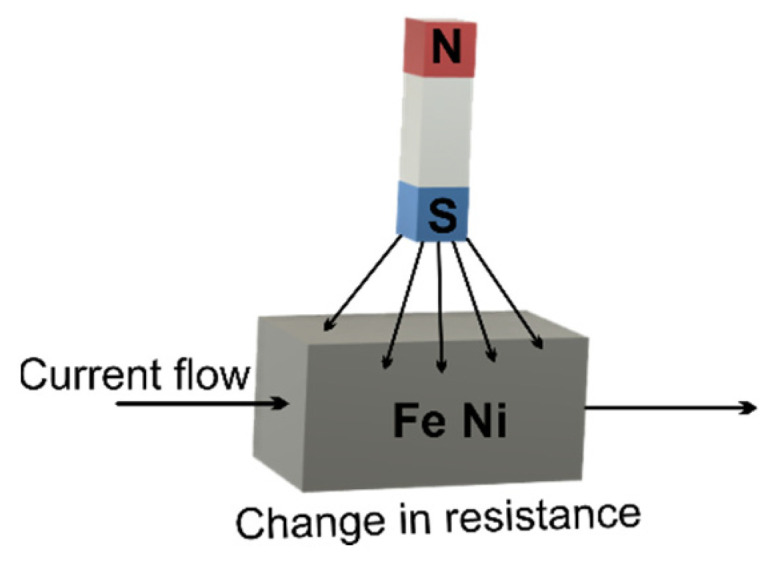
MEMS magnetometer—Magneto-resistive effect.

**Figure 4:**
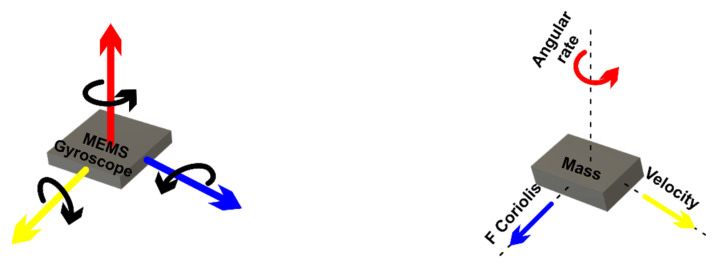
Gyroscope.

The following will describe the principle of the three sensors that makes up the absolute guidance module:

- *MEMS accelerometer*—this is an electromechanical device that converts mechanical force into an electrical signal. This device measures acceleration by measuring the change in capacity; the microstructure of this device can be seen in Figure 1. It has a mass attached to a screen that is limited to moving along a direction and a fixed outer plate, so that when acceleration is applied in a certain direction, the mass will move the capacity between the plates; therefore, this mass changes. This device can be used in civilian and military applications [23].

- *MEMS magnetometer*—the operation of this device is based on measuring the earth’s magnetic field using the Hall effect or resistive effect. In almost 90% of the cases, the sensors on the market work based on the Hall effect. In the case of sensors that use the Hall effect, electrons flow from one side of the board to the other side of the board (Figure 2a). If a magnetic field is brought close to the plate, the flow of electrons is disturbed, they are deflected and a division is made between negatives and positives poles (Figure 2b). If a multimeter is placed on both sides and a measurement is made, a voltage is obtained. The other 10% of sensors on the market use the magneto-resistive effect (Figure 3). These sensors use materials that are sensitive to the magnetic field and, are usually made of iron and nickel. When these materials are exposed to the magnetic field, they change their resistance [24].
- *MEMS gyroscope*—this device (Figure 4) measures the angular rate using the Coriolis effect when a mass moves in a certain direction at a certain speed, and when an external angular rate is applied, as shown by the yellow arrow, a force will appear (blue arrow) which will determine the perpendicular displacement of the mass; therefore, similarly to the accelerometer, this displacement will cause the change in capacity which will be a measurement process and will correspond to a certain angular rate [25].

**Figure 5:**
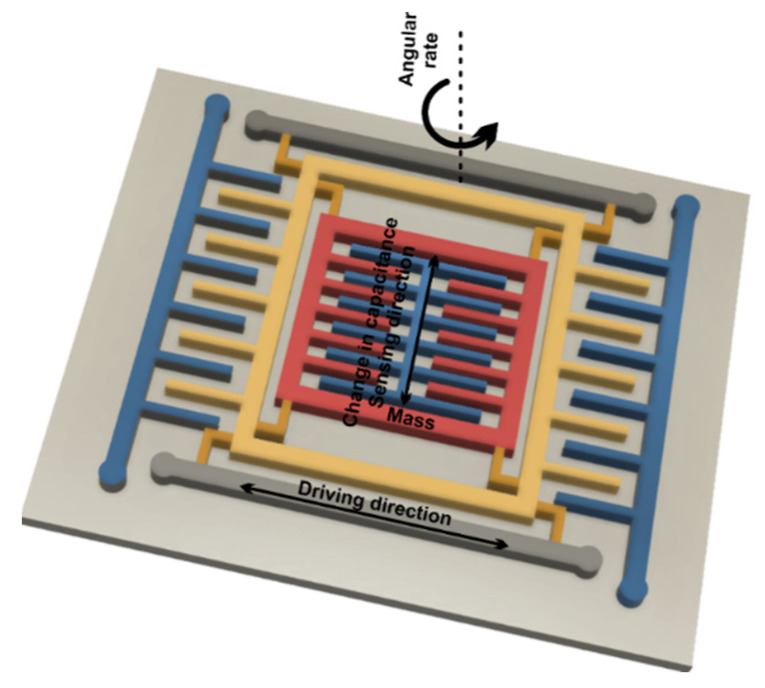
Microstructure of a gyroscope.

Figure 5 shows the microstructure of the gyroscope, a mass that is constantly moving or oscillating, and when an angular rate is applied, a flexible part of the mass will move and make a perpendicular motion.

## 4. Materials and Methods 

### 4.1. Components and Connections in the System

**Figure 6:**
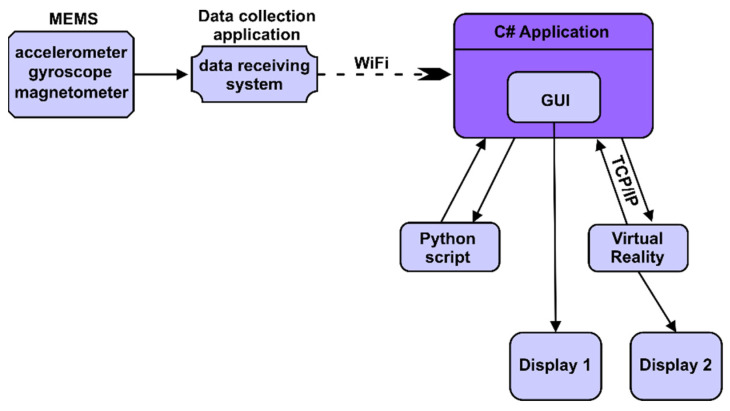
Interconnection of components.

In the block diagram in Figure 6 we can see an interconnection of the logic components in the system control. A nine-axis sensor is used to accurately position the drone. This type of sensor includes an accelerometer, a magnetometer and a gyroscope. An application that is on the ESP32 microcontroller (AI-Thinker, Shenzhen, China) collects data from the three sensors, after which these data are sent via the Wi-Fi protocol to the C# application that is on the computer. From here, through a user interface (GUI), these data are directed to be used. This user interface connects to a Python script that accesses the Keras 2.4.0 library [26] from TensorFlow 2.3.0, which includes artificial neural networks. This user interface also connects via the TCP/IP protocol to the virtual reality application to control it. Two monitors connected to the same computer in extended format are used to display the user interface of the C# application (made with C# version 7.3) and the virtual reality application.

**Figure 7:**
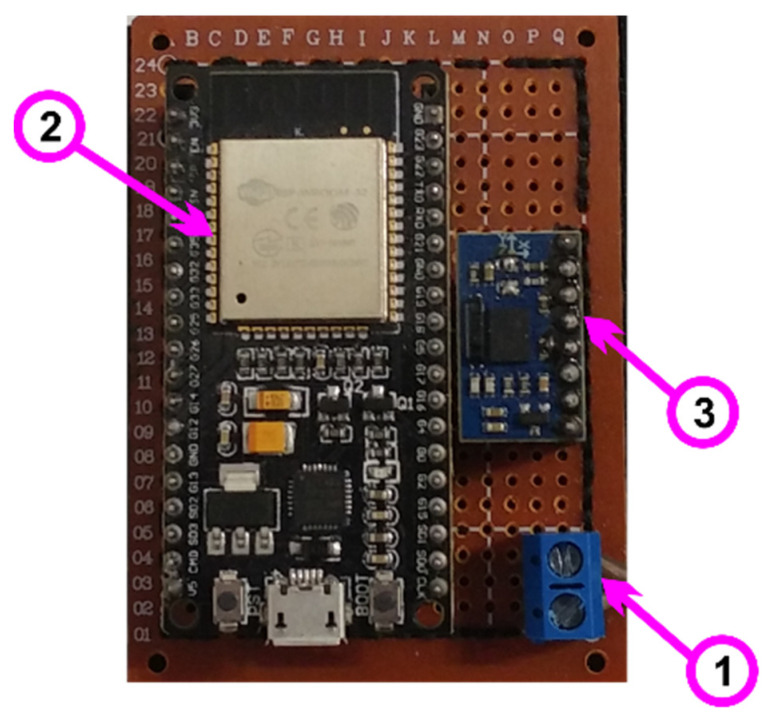
Device for receiving and transmitting data from sensors.

For the application created in the Arduino language that is on the microcontroller and which is used to retrieve data from the sensors, a device has been created that attaches to the upper limb of a person who is involved in the experiment, as can be seen in Figure 7. This device contains the following components:

(1) Device power supply: 5 V DC;
(2) Microcontroller ESP32, which is a development board that contains the following specifications:
  - 240 MHz microcontroller, dual-core—Tensilica LX6 (Texas Instruments, Dallas, TX, USA);
  - 520 KB SRAM memory;
  - Bluetooth dual mode (classic and BLE);
  - 16 MB flash memory;
  - Working temperature: −40 °C to 125 °C;
  - PCB antenna (has connector for external antenna);
  - Wireless transfer rates: 150 Mbps@11n HT40, 72 Mbps@11n HT20, 54 Mbp@11 g and 11 Mbp@11b;
  - 135 Mbps UDP connectivity;
  - Average operating current: 80 mA;
  - Wi-Fi: 802.11 b/g/n (802.11n up to 150 Mbps);
  - Bluetooth: Bluetooth v4.2 BR/EDR and BLE;
(3) Absolute orientation sensor—IMU BNO055 (Bosch Sensortec, Beijing, China), with the following characteristics:
  - Using chip: Original NEW BNO-055;
  - Built in nine-axis sensor and MCU resources;
  - Power supply: 3–5 v (internal low dropout voltage regulator);
  - Absolute orientation (Euler Vector, 100 Hz)—Orientation data on three axes based on a 360° sphere;
  - Absolute orientation (Quaternion, 100 Hz)—Four-point quaternion output for more accurate data handling;
  - Angular velocity vector (100 Hz)—Three axes for “rotational speed” in rad/s;
  - Acceleration vector (100 Hz)—Three axes for acceleration (gravity + linear motion) in m/s^2^;
  - Magnetic field strength vector (20 Hz)—Three axes for magnetic field strength detecting in micro Tesla (uT);
  - Linear acceleration vector (100 Hz)—Three axes for linear acceleration (acceleration minus gravity) in m/s^2^;
  - Vector for gravity (100 Hz)—Three axes for gravitational acceleration (minus any motion) in m/s^2^;
  - Communication mode: Standard IIC/serial communication protocol.

### 4.2. Software Application Development

The software application includes programs implemented in three programming languages, as follows: Arduino, C# and Python. The program implemented in the Arduino language is stored on the ESP32 microcontroller, a microcontroller that is part of the device that attaches to the upper limb of a person who controls the drone. The program on the ESP32 microcontroller takes the data from the three sensors (accelerometer, gyroscope and magnetometer) and encodes them in the format accepted by the C# application. As long as the Arduino script is connected to the C# application, if there are changes to the previously sent data, new encrypted data will be transmitted to the C# application.

**Figure 8:**
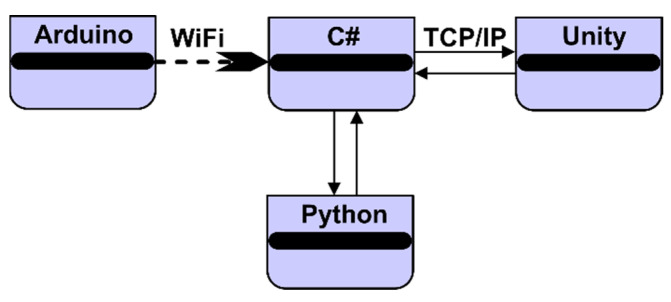
Software architecture.

The script implemented in Python [27] processes the data from the sensors using an algorithm specific to artificial intelligence [28]. The C# programming language [29] is used both for the implementation of the graphical user interface and for the development of the virtual application using the Unity development environment. The architecture of this software application that shows the communication between programs is shown in Figure 8.

### 4.3. C# Software Application

**Figure 9:**
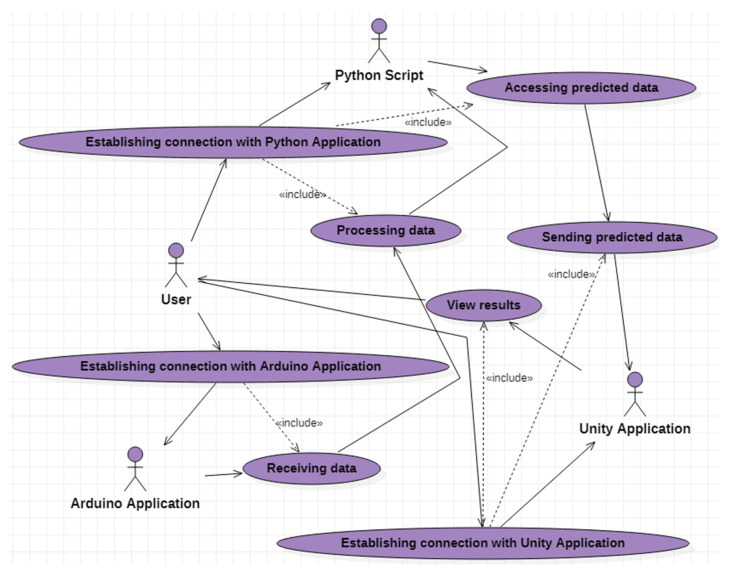
UML use cases diagram.

Using UML [30], the use case diagram shown in Figure 9 specifies all the functionalities of the application developed through the usage of C# programming language [31]. This UML diagram contains:

- eight use cases that describe the functionalities of the software application;
- four actors:
  ▪ the user who represents the external entity with which the C# application interacts;
  ▪ application implemented in Arduino;
  ▪ Python script;
  ▪ Unity application.
- Relations between the user and the use cases (association relations), as well as relations between the use cases (dependency relations).

The “Establishing connection with Arduino Application” use case allows the user of the C# application to connect to the application implemented in Arduino. After connecting, it will be possible to view the data received from the sensor through the functionalities associated with the “Receiving data” use case. 

The “Establishing connection with Python Application” use case offers the user of the C# application the possibility to connect to the application implemented in Python. After connecting to the Python application, due to the “Processing data” use case, the information received from the sensor is structured and sent to the intelligent mode in order to predict the data necessary for the drone control. The functionalities associated with the “Accessing predicted data” use case allow the data estimated to be used by the intelligent script, and then, through the “Sending predicted data” use case, sent to the virtual reality application to be used to establish the drone trajectory.

**Figure 10:**
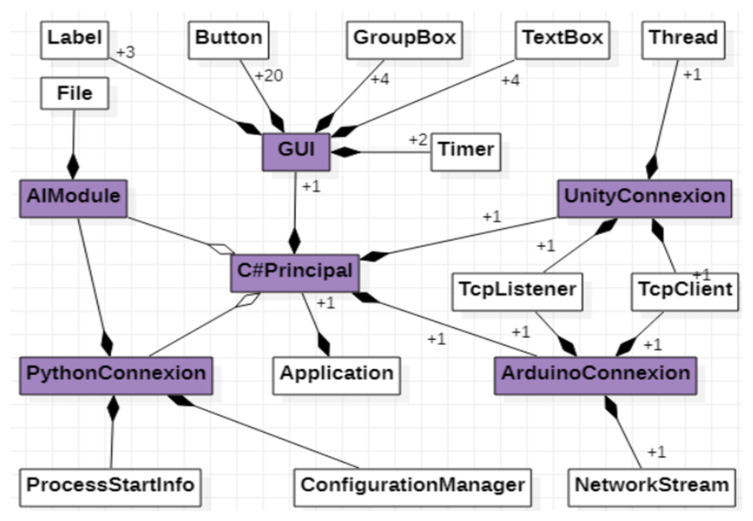
UML class diagram.

Conceptual modeling [32] allows the identification of the most important concepts for the computer system. Starting from the functionalities specified in the use case diagram, six classes have been designed and implemented, among which there are composition and aggregation relations [33], as shown in Figure 10. The six classes are:

- ***GUI*** class—allows interaction with the application user.
- ***ArduinoConnexion*** class—makes the connection with the application implemented in Arduino, allowing the taking over of the values from the sensor.
- Python, allowing both the transmission of input data to the artificial neural network [34] and the values predicted by the network.
- ***PythonConnexion*** class—makes the connection with the script implemented in 4.3.1. Graphical User Interface
- ***UnityConnexion*** class—makes the connection with the virtual application implemented in Unity.
- ***C#Principal*** class—represents the main class of the application, being composed of one object of each previously specified class.

#### Graphical User Interface

The user interface design was created in order to be able to control the drone from the virtual reality application. Through the user interface, the drone can be controlled manually with the help of buttons or automatically using the sensor module that was attached to the user’s upper limb. Here, the control is conducted using only data received from sensors or these data being passed through the neural network algorithm, and then these data are used to help control the drone.

**Figure 11:**
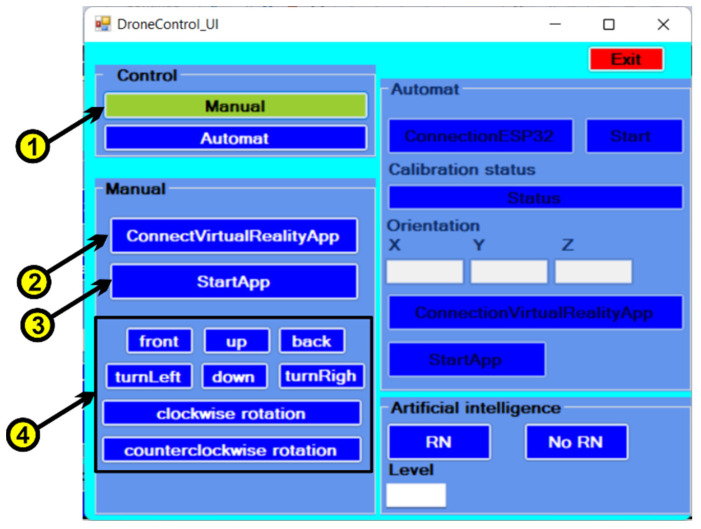
User Interface—manual control.

**Figure 12:**
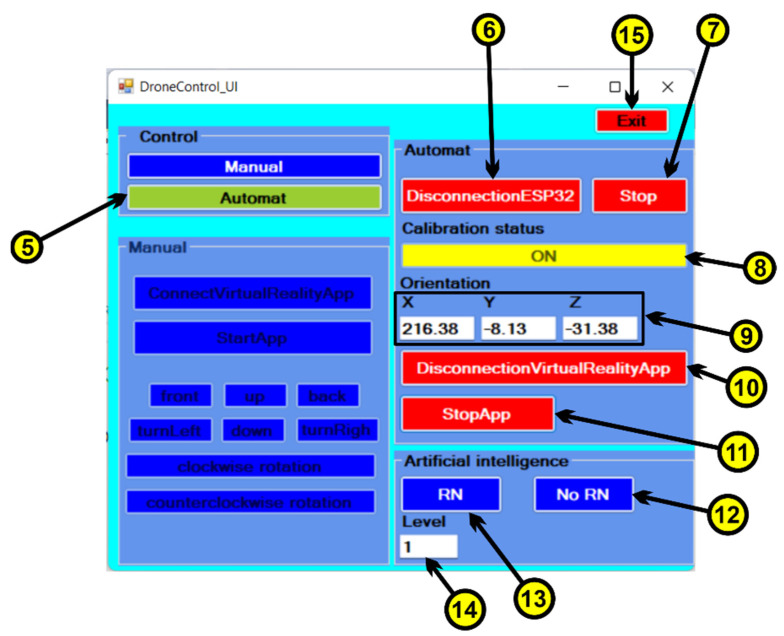
User Interface—automatic control.

Based on the features specified in the use case diagram, the graphical user interface was developed using the C# programming language and the Microsoft Visual Studio development environment. In order to be able to communicate between the data collection system from the sensors, the machine learning [35] module implemented in the Python programming language and the virtual reality application, a user interface is used (Figure 11). The following steps will be followed to use the user interface:

- The first time, we choose the type of control, which can be manual or automatic. For manual control, press the “Manual” button (Figure 11, 1), and at this moment a user interface is activated through which the drone can be controlled in manual control, the automatic control of the drone being inactive. The following commands are used for manual drone control:
  ▪ to begin, we must press on the “ConnectVirtualRealityApp” button (Figure 11, 2) to make the connection between the C# application that contains the user interface and the virtual reality application via the TCP/IP protocol;
  ▪ by pressing the “StartApp” button (Figure 11, 3) the data communication between the C# application and the virtual reality application begins;
  ▪ the following buttons (Figure 11, 4) control the drone, as follows:
  - when the “up” button is pressed, the drone rises in the vertical plane;
  - when the “down” button is pressed, the drone descends in the vertical plane;
  - when the “front” button is pressed, the drone moves forward in a horizontal plane;
  - to move the drone back horizontally, we must press the “down” button;
  - when the “turnLeft” button is pressed, the drone rotates counterclockwise;
  - when the “turnRight” button is pressed, the drone rotates clockwise;
  - via the “clockwise rotation” button, the drone will make a clockwise turn;
  - via the “counterclockwise rotation” button, the drone will make a counterclockwise turn.
  • Pressing the “Automatic” button (Figure 12, 5) deactivates the manual control interface (Figure 11) and activates the automatic drone control interface (Figure 12).

**Figure 13:**
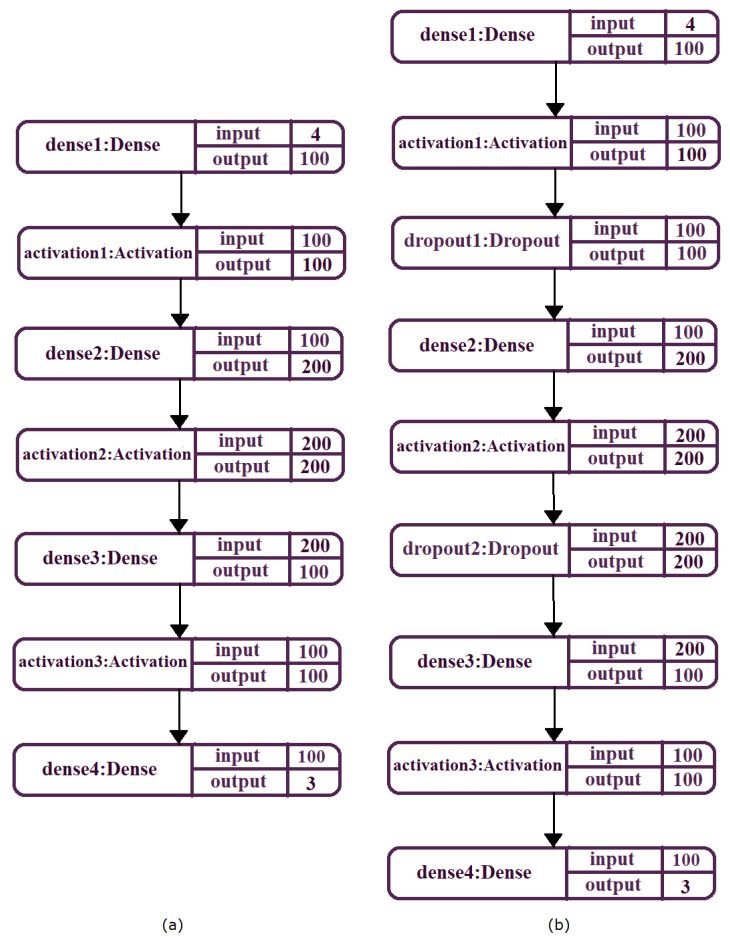
(**a**) LSTM architecture. (**b**) The modified MLP architecture.

**Figure 14:**
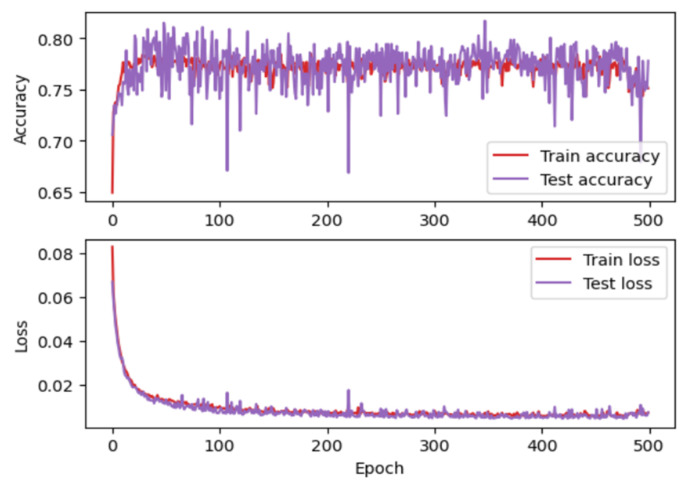
Accuracy corresponding to the first architecture.

The automatic drone control interface contains the following commands:

▪ by pressing the “ConnectionESP32/DisconnectionESP32” button (Figure 12, 6), a connection is made by Wi-Fi communication between the C# program containing the user interface and the Arduino program which is on the ESP32 microcontroller;
▪ pressing the “Start/Stop” button (Figure 12, 7) starts the data transfer via Wi-Fi communication between the C# application and the Arduino application;
▪ at the beginning of the use of the sensor device, the sensors must first be calibrated, and when the sensors have been calibrated, the status with the text “ON” on a yellow background is displayed on the user interface (Figure 12, 8);
▪ after the sensors have been calibrated, they can be used in the control to orient the drone on the X, Y and Z axes (Figure 12, 9);
▪ to connect via the TCP/IP protocol between the C# application and the virtual reality application, we must press the “ConnectionVirtualRealityApp/DisconnectionVirtualRealityApp” button (Figure 12, 10);
▪ to start the data transfer between the C# application and the virtual reality application, we must press the “StartApp/StopApp” button (Figure 12, 11);
▪ in order to control the drone directly through the sensor device that was attached to the upper limb, without using neural networks, the “No RN” button is used (Figure 12, 12);
▪ by pressing the “RN” button (Figure 12, 13), the data that are received from the sensors are first passed through the neural network algorithm, after which they are sent to the virtual reality application to help control the drone;
▪ the “Level” field (Figure 12, 14) is used to display the level traveled by the drone.

### 4.4. Arduino Software Application

The programming language used to write the control program found on the ESP32 microcontroller is Arduino, and the programming environment is an open-source program called Arduino Software (IDE). Prior to the implementation of the program in Arduino, the following libraries were configured in the development environment used:

- Wire.h: through this library, the communication of the IMU sensor BNO055 with the development board ESP32 is made through the IC2 protocol;
- WiFi.h: library used to connect the development board with microcontroller to the internet via the TCP/IP protocol;
- Adafruit_Sensor.h, Adafruit_BNO055.h: these libraries contain methods that are used in writing the program for obtaining data from the BNO055 sensor;
- utility/imumaths.h: library containing mathematical methods for inertial units of measurement.

In the implementation of the Arduino script, the connection is made with the main application implemented in C# through the TCP/IP protocol. While the connection is active, the data from the sensors are processed and then encoded and sent to the C# application with a frequency of 50 milliseconds. 

### 4.5. Python Software Application

For the prediction of the drone’s coordinates, we chose to use a multilayer perceptron [36] neural network. This network, classified in the ANN [37] feedforward category, consists of three or more levels distributed as follows:

- a level corresponding to the input data;
- a level corresponding to the output data;
- one or more hidden levels that represent the computational engine of the MLP [38] network.

With the exception of the first level nodes, the rest of the nodes are neurons that use an activation function. MLP is based on a supervised learning method named backpropagation for training. Its manifold layers and non-linear activation function differentiate MLP by a linear perceptron. It can discern data which are not separable from a linear point of view. Every layer is supporting the following layer through the outcome of their calculation, or their internal data representation. This procedure goes through all the hidden layers to the output one. Backpropagation represents the learning technique which affords the multilayer perceptron to iteratively adapt the weights from the network layers, with the purpose of optimizing the cost function. 

**Figure 15:**
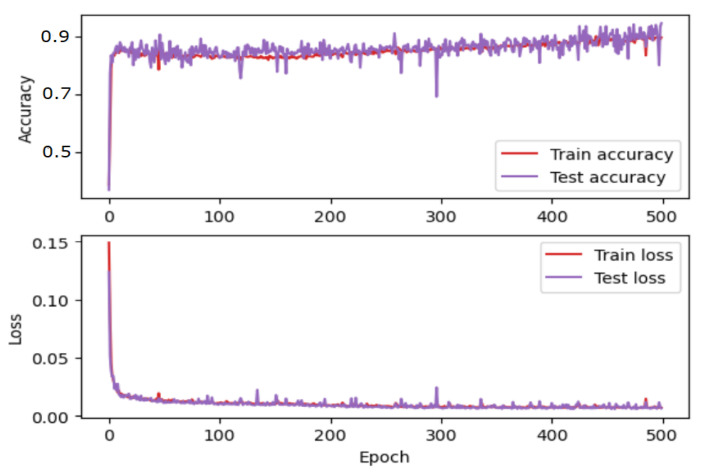
Accuracy corresponding to the second architecture.

From implementation of MLP, Python programming languages and four machine learning libraries were used: Keras [39], Tensorflow [40], MathPlotLib [41], and Sklearn [42]. The first layer receives 4 inputs (equal to the number of input characteristics) and returns 100 outputs. The second hidden layer receive 100 entries and returns 200 values. The third hidden layer receives 200 entries and returns 100 values. The last layer has three outputs which represent the estimated coordinates of the drone. This architecture is represented graphically in Figure 13a. After training this first MLP architecture on 500 epochs, the accuracy and the loss shown in Figure 14 were obtained. To improve the accuracy and loss values, the MLP architecture is updated by applying dropout layers. The modified MLP architecture is presented in Figure 13b. By training this new architecture for 500 epochs, an obvious improvement in accuracy and loss was obtained (Figure 15). 

### 4.6. Unity Application

At the development of the virtual reality application in the Unity environment, asset packages that were downloaded from the Unity Asset Store were used, but also components that were first designed in the Fusion360 program [x4]. It started first by adding a plan that created an environment using the following packages: Standard Assets, Fantasy Skybox FREE, Grass And Flower Pack, HQ_BigRock and Conifers. Once the environment was created, components that were created in the Fusion360 program were important in the Unity environment, these being the obstacles and the drone. Files in the C# programming language have been written for drone and view camera control. These files communicate with the user interface created in Visual Studio via the TCP/IP protocol.

**Figure 16:**
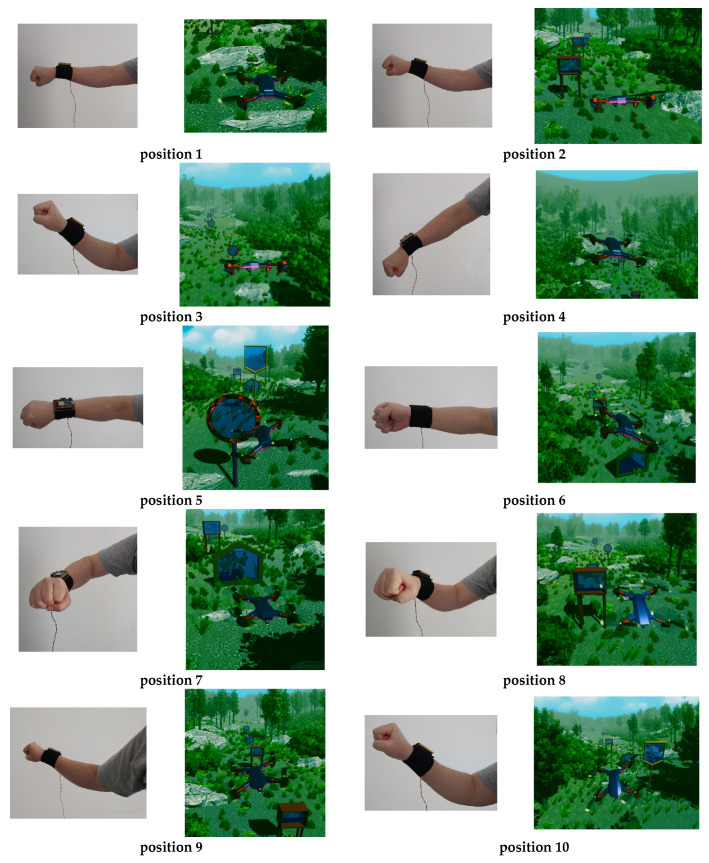
Drone control positions.

Description of the Operation of the Virtual Reality Application. Prior to launching the virtual reality application, the device containing the sensors is attached to the user’s upper limb, as shown in Figure 16. In the next step, we open the user interface and press the “Automatic” button (Figure 12, 5), and pressing this button activates the automatic control mode for drone control. To connect the sensor device with the C# application, we must press the “ConnectionESP32” button, and when the connection is made on the connect button, the message “DisconnectionESP32” appears (Figure 12, 6) because the connection button contains two states (one state for connection and a disconnect status). 

Continue with the press of the “Start” button (Figure 12, 7) and, from this moment, the transmission of the data taken from the sensors begins (Figure 12, 9). In order for the sensors to work properly, it is necessary to calibrate the sensors by movement the lower limb in different positions. After the calibration has been successfully completed on the user interface, the status “ON” appears on a yellow background (Figure 12, 8). Then, we click on the “ConnectionVirtualRealityApp” button (Figure 12, 10) to connect the user interface to the virtual reality application via the TCP/IP protocol. After the communication has been made, on the connect button, the message “DisconnectionVirtualRealityApp” appears (Figure 12, 10). Once the connection has been made, we must press the “Start” button (Figure 12, 11) to start the data coming from the sensors.

To control the drone, the user will need to perform the following upper limb movements in the following 10 positions, shown in Figure 16:

- position 1: the drone remains in a static position above the earth’s surface;
- position 2: moving the drone in the forward direction;
- position 3: lifting the drone vertically;
- position 4: lowering the drone vertically;
- position 5: rotating the drone in the counterclockwise direction;
- position 6: rotating the drone in the clockwise direction;
- position 7: turning the drone in the counterclockwise direction;
- position 8: moving the drone in the forward direction;
- position 9: turning the drone in the clockwise direction;
- position 10: moving the drone in the forward direction.

**Figure 17:**
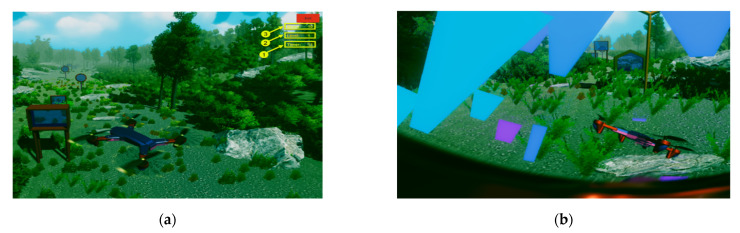
(**a**) Drone control for obstacles crossing. (**b**) Passing the drone through obstacles.

Next, the operating mode for drone control is chosen. By pressing the “No RN” button (Figure 12, 12), a drone control is chosen without involving the neural network part. Here, the user, by moving the upper limb, controls the drone to travel the obstacle course only based on data from the sensors. When the “RN” button is pressed (Figure 12, 13), the data from the sensors are sent to the neural network for processing, and after being processed, they are transmitted to the virtual reality application to help control the drone by predicting the positions where there are obstacles that the drone must travel through. From this point on, the user can start the drone control process for the obstacle course. For level 1 (Figure 17a, 2), the user has go through 10 obstacles (Figure 17a, 2) in a maximum of 120 s, and a timer has been created for this in the virtual reality application (Figure 17a, 1), which starts automatically when the user starts the virtual reality application. The obstacle that the drone has go through contains glass and, when the drone passes, it collides with the glass and it breaks (Figure 17b), thus making a count of the obstacles passed.

### 4.7. Experimental Validation

#### 4.7.1. Participants

Eight healthy subjects (6 men, 2 women with a mean age of 40 years) participated in the experimental study after giving informal written consent. All participants who performed the experiment used the dominant (right) upper limb to control the drone. Six of the subjects did not know the intent of the experiment and had no practice of controlling the drone using the device that was attached to the upper limb. The demographic details of the participants are presented in Table 1.

#### 4.7.2. Performance Evaluation

For each combination of dominant upper limb movement, the performance of each participant in controlling the drone is analyzed. To control the drone, each participant involved in the experiment took turns attaching the sensor device to their upper limb. After making the necessary settings on the user interface (Figure 12) to use the sensor device, in the next step, the sensor was calibrated by a few upper limb movements directed on the axes Ox, Oy and Oz. After the calibration has been performed, from the application implemented in Arduino is sent to the C# application a value that confirms the end of the calibration. On the graphical user interface, this calibration will be confirmed by setting the “ON” button on a yellow background (Figure 12,8). After the virtual reality application is opened, and to start the virtual reality application, the user must click on the “StartApp” button (Figure 12, 11) on the user interface. 

**Figure 18:**
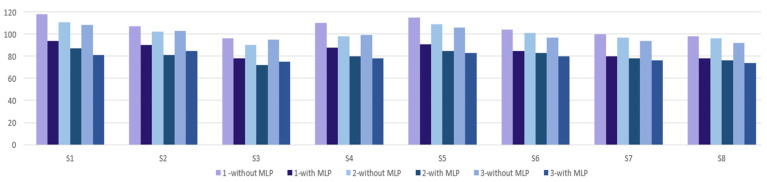
Comparing results.

Participants included in the experiment took turns controlling the drone to traverse the obstacles in the virtual reality environment in a set time period (Figure 17, 1). Subjects who participated at the experiment at Level 1 had to complete ten obstacles before the stopwatch reached zero seconds. In the first stage, they did not use the artificial neural network in the control of the drone, this being used in the second stage. Figure 18 shows the times obtained by the eight subjects involved in the experiment for three repetitions of the route, in the situation where the neural network was not used in the control of the drone and the situation in which the artificial neural network was used to control the drone. Analyzing this graphic, it can be seen that time required to cover the route is shorter when the neural network is used for each subject.

## 5. Conclusions

This article describes a study of drone control using a new device developed by the authors. It allows precise control due to the combination of MEMS sensor technology and the MLP artificial neural network. This device allows for control of a drone in a virtual reality simulator. In order to control the drone, the person involved in this experiment will have to have this device attached to his upper limb, and the positioning of the drone is achieved by moving the upper limb. For an interaction between the virtual reality simulator and the user, a graphical interface has been developed using the Visual Studio development environment. In this simulator, the drone can be controlled either with the raw values received from the sensors or with the predictions provided by the intelligent module. From the last two figures presented in the article it was found that users achieve the right goal in less time when using the intelligent module for drone control. As a further development, it will be desired to implement the intelligent module using other artificial intelligence techniques and compare them, so that the accuracy of the prediction in drone control is as close to 100% as possible.

## Figures and Tables

**Table 1:** This subject-specific demographics and anatomic measures.

Subject	Age	Gender	Forearm Length (in mm)
1	31	m	265.50
2	27	f	270
3	35	m	260
4	42	f	260
5	41	m	270.50
6	42	m	270
7	36	m	260
8	30	m	250
